# MicroRNAs Involved in Anti-Tumour Immunity

**DOI:** 10.3390/ijms14035587

**Published:** 2013-03-11

**Authors:** Hong W. H. Yu, Daniel M. Y. Sze, William C. S. Cho

**Affiliations:** 1 Department of Health Technology and Informatics, the Hong Kong Polytechnic University, Hong Kong, China; E-Mail: daniel.sze@polyu.edu.hk; 2 Department of Clinical Oncology, Queen Elizabeth Hospital, 30 Gascoigne Road, Kowloon, Hong Kong, China; E-Mail: chocs@ha.org.hk or williamcscho@gmail.com

**Keywords:** anti-tumour, immunity, microRNA

## Abstract

MicroRNAs (miRNAs) are a category of small RNAs that constitute a new layer of complexity to gene regulation within the cell, which has provided new perspectives in understanding cancer biology. The deregulation of miRNAs contributes critically to the development and pathophysiology of a number of cancers. miRNAs have been found to participate in cell transformation and multiplication by acting as tumour oncogenes or suppressors; therefore, harnessing miRNAs may provide promising cancer therapeutics. Another major function of miRNAs is their activity as critical regulatory vehicles eliciting important regulatory processes in anti-tumour immunity through their influence on the development, differentiation and activation of various immune cells of both innate and adaptive immunity. This review aims to summarise recent findings focusing on the regulatory mechanisms of the development, differentiation, and proliferative aspects of the major immune populations by a diverse profile of miRNAs and may enrich our current understanding of the involvement of miRNAs in anti-tumour immunity.

## 1. Introduction

MicroRNAs (miRNAs) are a group of small non-coding RNA molecules that play a central role in a number of biological processes through the post-transcriptional regulation of gene expression. This class of molecules provides the relevant regulation epigenetically in addition to the major categories of DNA methylation, histone deacetylation, chromatin remodelling, gene imprinting, and noncoding RNA regulation [1].

miRNAs have been shown to be critical contributors in the pathogenesis of many diseases including cancer. On one hand, miRNAs present as potential future diagnostic and prognostic markers and as viable therapeutic targets for cancer treatment [2–4]. On the other hand, miRNAs are also important regulators of the development and functions of diverse immunologically important cell types and the related complex cytokine network.

There are two major arms of the immune system, known as the innate and adaptive immune responses, which work in a complementary manner to help the body maintain its healthy status. The major players of the innate immune system that provide the first line of defence are natural killer (NK) cells, γδ T cells and macrophages. These cells, together with some inflammatory cytokines, critically defend the barriers at the mucosal and cutaneous levels. In the adaptive immune system, specificity and memory are the two key characteristics that are absent in the innate immune system. Dendritic cells (DC), B cells and T cells are the three cooperating major cell types that constitute the adaptive immune system.

**Figure 1:**
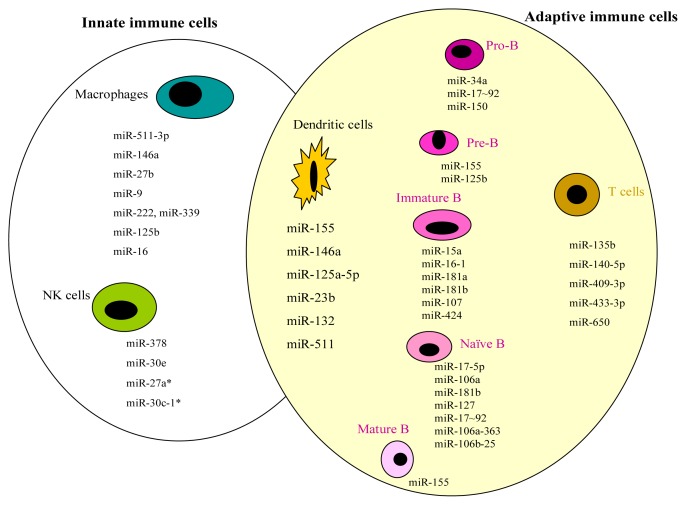
Involvement of miRNAs in immune cells.

Some lineage-specific miRNAs have been found to play a key role in regulating various developmental stages of the lineage development of the two major arms and thus affect different cell types in the mature state and their progenitor counterparts [5]. miRNAs are also involved in the activation of various immune cell components and thus regulate various cancer-related inflammatory responses and cytokine signalling [6,7]. Furthermore, certain miRNAs display the important modulatory capabilities on the life span, migration and immunogenicity of immune cells. For example, research showing that harnessing these features of miRNA targeting of DCs may lead to possible DC vaccine development for future clinical trials [8]. Figure 1 forms a visible representation of the involved miRNAs in immune cells.

## 2. miRNAs and Innate Immunity

One of the important cell types for the first line of defence of the immune system is the epithelial cell population that expresses some pathogen pattern recognition receptors, including the Toll-like receptors (TLRs), which recognise pathogen-associated molecular patterns and can induce strong pro-inflammatory responses [9]. In the recognition of pathogens, TLRs recruit adaptor proteins to facilitate the activation of downstream signalling cascades, such as the nuclear factor kappa B (NF-κB) and mitogen-activated protein kinase pathways. The activation process induces the expression of adhesion molecules, inflammatory mediators of cytokines or chemokines, and antimicrobial peptides that initiate innate immune responses and anti-tumour involvement, which are regulated by a number of complex networks. miRNAs are the essential portion of these complex regulatory networks for the cellular processes, differentiation and final fate of these innate immune cells [10].

Another major cell population of the innate immune response is the macrophages. Tissue macrophages detect pathogens through TLRs and after phagocytosing these pathogens, initiate the innate immune responses [11]. Macrophages, in response to microbes, produce cytokines that stimulate inflammation via leukocyte recruitment. Subsequently, NK cells are activated and produce the macrophage-activating cytokine IFN-γ, which is now known to have a central role in the regulation of inflammation. Additionally, miR-19 has been found to be involved in modulating this NF-κB-based inflammatory activity [12]. Table 1 provides a summary of a list of miRNAs and their corresponding target genes in the activation of macrophages and NK cells.

### 2.1. Macrophages

Macrophage subsets include the ‘classically activated’ pro-inflammatory (M1) and ‘alternatively activated’ anti-inflammatory (M2) cells [23]. The transcriptional activation of macrophage mannose receptor 1 (MRC1) in M2-polarised tumour-associated macrophages (TAMs) involves miR-511-3p regulation to establish the threshold for inflammatory cell activation in tumours in which the induced expression of miR-511-3p downregulates the pro-tumour gene signature of MRC1(+) TAMs and inhibits tumour growth [13]. The transcription of the miR-146a gene in human THP-1 cells has been reported in response to TLR-4 signalling, and miR-146a acts on the target genes TNF-receptor associated factor 6 (TRAF6) and IL-1 receptor associated kinase 1 (IRAK1) through the NF-κB-dependent cascade [14]. Similarly, miR-27b expression can also be induced directly by lipopolysaccharide (LPS) [15]. In addition, LPS will regulate miR-9 expression via the myeloid differentiation primary response gene 88 through the NF-κB pathway in human macrophages and neutrophils [16].

miRNAs, such as miR-222 and miR-339, have also been implicated in the expression of adhesion and costimulatory molecules, including the intercellular adhesion molecule 1 (ICAM1), which is essential in the interactions of innate immune cells [17]. miR-125b has also been reported to regulate the expression of cytokines and chemokines by targeting and suppressing tumour necrosis factor α (TNF-α) transcription in mouse Raw 264.7 macrophages [18]. Furthermore, miR-16 has been demonstrated to be one of the critical regulators of inflammatory responses, with a high level of miR-16 in inflammatory cells that inhibit the production of inflammatory mediators [19].

### 2.2. NK Cells

NK cells are essential innate immune components with potent cytotoxicity and type I IFN (INF-α) initiation capability. Wang *et al.*[20] reported that the expression of miR-378 and miR-30e is markedly decreased in IFN-α-activated NK cells that are labelled as granzyme B (GZMB)- and perforin (PRF)-positive. In contrast, downregulated miR-378 and miR-30e are the negative regulators of NK cell cytotoxicity during activation in the subset of sorted CD16(+)CD56(dim)CD69(+) human NK cells.

Another important cytokine produced by NK cells is TNF-α. The up-regulation of miR-30c-1* is now known to trigger the overexpression of transmembrane TNF-α, which in turn enhances NK cell cytotoxicity against the hepatoma cell lines SMMC-7721 and HepG2 by targeting the transcriptional repressor gene HMBOX1 [22,24]. In contrast, miR-27a* is able to negatively regulate NK cell cytotoxicity by silencing the genes PRF1 and GZMB, as shown by the knockdown of miR-27a* in NK cells, which leads to decreased tumour growth in a human tumour xenograft model [21].

## 3. miRNAs and Adaptive Immunity

miRNAs play an important role in the early differentiation of B cells and act as regulators of the immune response by T cells and DCs in adaptive immunity. Great efforts have been made to demonstrate the role of miRNAs in adaptive immune cells in recent years. Table 2 summarises the miRNAs involved in adaptive immunity and their respective target genes, with the third column showing those miRNAs participating in these cell types at various developmental stages.

### 3.1. B Cells

The expression of the transcription factors for B-cell development has been found to be precise and time-specific under the influence of a few miRNAs on B cells at various maturation stages. It is evident that the alteration of miRNA expression may lead to important functions in cellular differentiation and be associated with the activation status of the mature B cells in the immune system [49]. The role of miRNAs in B-cell development and B-cell lymphogenesis is largely unknown. The stage-specific expression of various miRNAs has suggested highly specialised regulatory functions in B-cell biology, which would reveal the cell type-specificity of miRNAs in B lymphocytes [50].

The expression of miR-150 was shown to block the transition from the pro-B to pre-B stage, likely through the down-regulation of c-MYB [28–30]. miR-17~92 is also essential for B-cell development, in which the absence of miR-17~92 will lead to the elevated expression of the pro-apoptotic protein BIM, which in turn inhibits B-cell development at the pro-B to pre-B transition. A link between the oncogenic properties of miR-17~92 and its physiological functions during B lymphopoiesis has been suggested [26]. Accordingly, B-cell development could be partially rescued by the ablation of BIM through the suppression of miR-17~92 [27]. In addition to miR-150 and miR-17~92, miR-34a was also found to block B-cell development at the transition from pro-B to pre-B cells via the target gene forkhead box transcription factor (FOXP1), which is a transcription factor gene; this process leads to a reduction in mature B cells. Accordingly, the knockdown of miR-34a resulted in the elevation of FOXP1 expression and an increase in mature B cells [25].

The association of miR-155 with the primary transcript of the host gene BIC was observed in preleukaemic pre–B-cell proliferation in the spleen and bone marrow of transgenic mice [31,32]. The high expression of miR-155 was also observed in murine B-cell precursors of acute lymphoblastic leukaemia or high-grade lymphoma, which were preceded by polyclonal pre-B cell proliferation. miR-155 directly targeted SRC homology 2 domain-containing inositol-5-phosphatase (SHIP) and CCAAT enhancer-binding protein beta (C/EBPβ), which are both regulators of the IL-6 signalling pathway. miR-155 was hypothesised to cause accumulation of large pre-B cells and acute lymphoblastic leukaemia by down-regulating SHIP and C/EBPβ [33].

The over-expression of miR-21 has been found in a number of tumour types. Medina *et al.*[51] demonstrated that the over-expression of miR-21 leads to a pre-B malignant lymphoid-like phenotype and that the tumours regressed completely in a few days after miR-21 was inactivated. miR-125b has been found to up-regulate a number of common myeloid progenitors and to inhibit the development of pre-B cells by acting on some candidate targets, including the induced pluripotent stem cell gene LIN28A [34]. miR-125b physiologically regulates haematopoietic development. LIN28A was saliently suppressed in mouse haematopoietic stem cells and progenitor cells, and the knockdown of LIN28A can lead to haematopoietic lineage skewing with an increase in myeloid cells but decrease in B cells.

The deregulation of miR-181a, miR-181b, miR-107 and miR-424 was found to lead to the subsequent overexpression of the oncogenic transcription factor pleomorphic adenoma gene 1 (PLAG1) in a number of chronic lymphocytic leukaemia (CLL) cases [36,37]. CLL is characterised by the clonal expansion of immature CD5-positive B cells; up to 20 percent of the patients with CLL are not controlled with standard therapies using cytotoxic agents. The 13q14.3 chromosomal region often found in patients with CLL contains miR-15a and miR-16-1 [35]. Tan *et al.*[38] characterised the miRNA expression profile of normal B cell subsets that included naïve, germinal centre (GC) B cells and memory B cells with a miRNA *in situ* hybridisation technique. Several miRNAs were elevated in GC B cells, such as miR-17-5p, miR-106a and miR-181b, whereas the gradual decrease in the staining intensity of these three miRNAs from the dark to light zone was observed in GC. miR-150 was the most abundant in all three B-cell subsets.

In a study of the expression of a panel of 15 miRNAs in some DLBCL cases, the expression of miR-17-5p was significantly higher in central nervous system diffuse large B cell lymphoma (DLBCL) than in testicular DLBCL, and miR-127 was found to be highly expressed in testicular DLBCL compared with central nervous system DLBCL [39].

Iqbal *et al.*[40] identified a 19-miRNA classifier including 6 up-regulated miRNAs and 13 down-regulated miRNAs that helped distinguish mantle cell lymphoma (MCL) from other aggressive lymphomas, and some of the up-regulated miRNAs were highly expressed in naïve B cells. The high expression of miR-17~92, miR-106a-363 and miR-106b-25 was observed in some patients with MCL in their studies. For example, miR-155 is encoded in the B cell integration cluster, and its knockdown leads to B cell defects and a failure of immunoglobulin-switched plasma cells. The transcription factor PU.1 is the validated target of miR-155 [41].

#### Related Signalling Pathways in Lymphoma

The PI3K/PTEN/AKT pathway is one of the key signalling pathways involved in the regulation of cell growth. The frequent dysregulation of the PI3K/PTEN pathway in human cancer demonstrates that this pathway is an appropriate target for cancer therapeutics [52]. Hafsi *et al.*[53] suggested that the dysregulated signalling of this pathway might be associated with activating mutations in PI3K-related genes. An increased PI3K signal will stimulate downstream AKT signalling, promote growth factor-independent growth and facilitate cell invasion and metastasis, which account for 50% of all human malignancies [54]. A common secondary genomic alteration detected in MCL is chromosome 13q31-q32 gain or amplification, which targets the miR-17~92 cluster. Rao *et al.*[55] demonstrated that the protein phosphatase PHLPP2, an important negative regulator of the PI3K/AKT pathway, was a direct target of miR-17~92 which also targeted PTEN and BIM. The inhibition of miR-17~92 suppressed the PI3K/AKT pathway, which in turn inhibited tumour growth in the xenograft MCL mouse model. Hence, targeting the miR-17~92 cluster may provide a novel therapeutic approach for patients with MCL.

### 3.2. T Cells

miRNAs have been shown to be involved in the regulation of T-cell responses, with a dynamic expression pattern relative to the various stages of T cell development. miR-135b was reported to mediate nucleophosmin-anaplastic lymphoma kinase (NPM-ALK)-driven oncogenicity and induce the immunophenotype of IL-17 in anaplastic large cell lymphoma (ALCL). Oncogene NPM-ALK strongly promoted miR-135b expression through the activation of transducer and activator of transcription (STAT) 3, and the elevated miR-135b targets FOXO1, a transcription factor regulating gluconeogenesis and glyconeogensis via insulin signalling in ALCL cells. Chemosensitivity in Jurkat cell line was found to be decreased by miR-135b [42]. Furthermore, miR-135b suppresses the T-helper 2 regulator gene STAT6 and GATA3, whereas antisense-based miR-135b inhibition induces tumour angiogenesis *in vivo*.

NKG2D, encoded by the KLRK1 gene, is one of the activating immunoreceptors found on CD8 T cells and NK cells, and its ligands are stress-inducible proteins, including ULBP1, that enable the recognition and lysis of tumour cells. Some miRNAs were shown to be involved in the post-transcriptional regulation of ULBP1 expression in Jurkat and HeLa cells. Among these miRNAs, miR-140-5p, miR-409-3p, miR-433-3p and miR-650 are involved in the regulation of ULBP1 expression [43].

### 3.3. Dendritic Cells

DCs are found in almost all peripheral tissues and in primary and secondary lymphoid organs. The antigen presentation of DC controls immunity and tolerance, is linked with almost all types of immune cells and plays major roles in regulation of immune responses [56].

Cubillos-Ruiz *et al.*[44] took advantage of the spontaneous enhanced endocytic activity of ovarian cancer-associated DCs to selectively supplement the immunostimulatory miR-155. Modulating the activity of miRNAs may lead to cancer interventions. miR-155 that has been processed endogenously would favour Argonaute 2 (AGO2) and AGO4 loading, resulting in transcriptional changes that might silence multiple immunosuppressive mediators [57]. Thus, tumour-infiltrating DCs were transformed into highly immunostimulatory cells, triggering potent anti-tumour responses to abrogate the progression of ovarian cancer.

Concerning the tolerogenic property of DCs, miR-23b may be one of the entry points in targeting the therapeutic management of allergies. The upregulation of miR-23b could be observed in bone marrow DCs (BMDCs) by ovalbumin in a murine model. Increased IL-10 levels, decreased IL-12 levels and an enhancement of the FOXP3^+^ CD4^+^ T regulatory cell differentiation were shown in BMDCs by the transfection of miR-23b, likely through the inhibition of the transmembrane protein family member NOTCH1 and the NF-κB signalling pathway [45]. Similar results were also obtained in human monocyte-derived DCs.

Some miRNAs, such as miR-146a and miR-155, may act as checkpoints in the cellular differentiation aspect of the immune system [46]. Using a miRNA array, Holmstrom *et al.*[8] demonstrated a significant induction of miR-155, miR-146a and miR-125a-5p in some donor DCs treated with LPS. In addition to miR-146a and miR-155, miR-132 is a TLR ligand-induced regulator of inflammatory mediators, which may modulate TLR pathway activation and may be used to develop relevant therapeutics for inflammatory diseases [47]. miR-511 has been identified as a novel potent modulator of the human immune response through the validation of CD80 expression and inhibition of miR-511 in HEK293 cells. Similarly, DC-specific intercellular adhesion molecule-3-grabbing non-integrin (DC-SIGN) was found to be reduced, thereby revealing that miR-511 may act as a positive regulator of TLR-4 [48].

## 4. miRNAs Related to Anti-Tumour Immunity

Cancer, as a result of a complicated multi-step process, involves the accumulation of sequential genomic alterations, including those of miRNAs, and can be characterised by uncontrolled proliferation, invasion and metastasis [58]. Increasing evidence shows an association with the aberrant miRNA expression patterns in human cancers [59,60]. miRNAs play crucial roles in the initiation and progression of human cancer [61], which in turn suggests their potential diagnostic and therapeutic roles in anti-tumour immunity. Table 3 summarises the altered expression of miRNAs with their respective target genes in various types of cancer.

Cancers are the major causes of mortality and morbidity in industrialised countries. Some concepts related to the mechanisms of the modulation of both innate and adaptive immune cells leading to anti-tumour effects will contribute to the regulation of carcinogenic processes and associated inflammatory effects. The strong balance between anti-tumour immunity and proinflammatory activity caused by the tumour in the tumour microenvironment niche may weaken anti-tumour immunity. The modulation of immune cells targeted for therapeutic intervention in malignant diseases may restore the sensitivity of cancer cells to chemotherapies. miRNAs should be analysed with nuclear factors, such as NF-κB, that serve as molecular links between inflammation and tumour progression [96].

With an increasing number of studies showing that miRNAs could function as oncosuppressors, the exploration of miRNA-based anticancer therapies was conducted to improve the response to current targeted cancer treatments. This improvement will provide the essential enhancement of the capability of targeting multiple effectors in pathways involving cancer cell proliferation and survival [97].

Some malignant diseases are developed in association with tumour viruses, and a number of studies have illustrated miRNAs as critical regulators of tumour pathways in which the dysregulation of cellular miRNA expression could promote tumour formation. Tumour viruses encode their own miRNAs, which manipulate the expression of cellular miRNAs to modulate the host cellular environment and in turn facilitate their respective infection cycles. The modulation of miRNA expression might influence the signal transduction cascades that favour tumourigenesis [98].

### 4.1. CNS Involvement

Dicer-mediated expression of miR-222 and miR-339 demonstrated the promoting effect on the immunoresistance of cancer cells through the regulation of the intercellular adhesion molecule ICAM1. miR-222 and miR-339 were found to be specifically down-regulated by the alteration of dicer expression in both colorectal and glioblastoma cell lines [17], suggesting novel methods to develop miRNA-targeted therapies to enhance the cytolysis of tumour cells in a variety of malignancies, including glioblastoma [62].

### 4.2. Cancer in Blood Components

miRNA expression has been found to be associated with the clinical outcome of patients and with drug resistance, which was described in B-CLL, lung cancer [99] and B-ALL [100]. The potential anti-tumour activity of IL-23 in paediatric B-acute lymphoblastic leukaemia (B-ALL) cells was investigated. The up-regulation of IL-23 dampened the tumour growth *in vitro* and *in vivo* through the inhibition of tumour cell proliferation and the induction of apoptosis, which was found to be associated with the IL-23-induced up-regulation of miR-15a expression and the consequent down-regulation of BCL-2 protein expression in paediatric B-ALL cells [65]. A similar study showed the anti-tumour effect of IL-27 against paediatric B-ALL cells with the down-regulated expression of miR-155 [66].

ALL and AML were demonstrated to have different miRNA expression patterns [63]. Moreover, miR-128a and miR-128b were highly expressed in ALL cells, whereas miR-223 and let-7b were detected in AML, which seemed to be significant and discriminatory for these leukaemias. Notably, 50% of known human miRNAs were found to be located at fragile sites and genomic regions involved in cancer, and these miRNAs might function as tumour suppressors or oncogenes [101]. The deregulation of miRNAs was observed in B-ALL, which appears to be one of the key mechanisms involved in B-ALL leukaemogenesis, which led to the suggestion that miRNAs represent new potential therapeutic targets [102]. In addition, miR-19 promotes leukaemogenesis in NOTCH1-induced T cell acute lymphoblastic leukaemia *in vivo* by regulating PTEN and BIM [64].

### 4.3. Gynaecological Cancers

miRNAs are involved in gynaecological disorders affecting the ovary or uterus, frequently affected by endometriosis, which is classified as a tumour lesion, and in malignant gynaecological diseases including endometrial, cervical and ovarian cancers. Emerging evidence has shown that deregulated miRNA expression might be involved in the multifactorial and polygenic diseases of endometriosis and that miRNAs appear to be potent regulators of gene expression in endometriosis, resulting in the prospect of using miRNAs as biomarkers or therapeutic measures for these cancers [103].

A significant decrease in the expression of miR-424 was observed in a number of cervical cancer tissue samples, which was positively correlated with poor prognostic clinicopathological parameters. The tumour suppressive role of miR-424 in the progression of cervical cancer via the up-regulated expression of CHK1 and p-CHK1 suggested miR-424 as a probable anticancer therapeutic target for cervical cancer patients [70]. Similarly, down-regulated miR-375 might contribute to the progression of cervical cancer based on the findings that miR-375 promoted cell malignant transformation via its target gene, SP1 [71]. Qiang *et al.*[72] also found that undermining miR214 expression in cervical cancer tissue and HeLa cells would insufficiently inhibit the probable oncogene plexin-B1, which might contribute to cervical tumour metastasis and invasion.

### 4.4. Cancers of the GI Tract

miR-206 was found to be significantly down-regulated in laryngeal squamous cell carcinoma (LSCC) tissue, and its transfection decreases the expression of vascular endothelial growth factor (VEGF) in LSCC cells, contributing to the tumour suppression function of miR-206 [73].

The high level of expression of miR-92 [74] and overexpression of miR-25 [75] were demonstrated in oesophageal squamous cell carcinoma (ESCC) tissues, and both miRNAs modulate the migration and invasion of ESCC cells. The expression of miR-92 and miR-25 was correlated with the status of lymph node metastasis, most likely through the repression of the CD324-induced expression of the tumour suppressor CDH1 gene but not with the apoptosis and proliferation of ESCC cells. Another study showed the high expression of miR-142-3p in ESCC cells, which was well correlated with the poor prognosis of ESCC patients [76]. miR-21, which had been recognised as an oncogenic miRNA in various malignancies, might also be one of the oncogenic miRNAs involved in ESCC [77]. miR-145, miR-133a and miR-133b were also revealed to have tumour suppressive effects inhibiting ESCC cell proliferation and invasion via repression of the FSCN1 gene, whereas FSCN1 was known to be involved in the metastasis of multiple tumour types and regulated by a number of miRNAs [78].

A relatively higher expression of miR-21 was exhibited in gastric cancer tissue. miR-21 might be involved in the initiation and development of gastric cancer by regulating the PTEN expression level [79]. miR-223 was found to be highly expressed in gastric cancer tissues and the corresponding gastric mucosal tissues, specifically in patients with lymph-node metastasis or metastatic disease at the advanced pathological M1 stage, in which the expression of the target FBXW7/hCDC4, a general tumour suppressor gene, was inversely correlated in the study [80]. The significantly down-regulated expression of miR-335 was confirmed in 4 gastric cell lines and 70 gastric cancer tissues, and elevated miR-335 was verified to suppress gastric cancer invasion and metastasis *in vitro* and *in vivo*, most likely via targeting the transcription factor gene SP1 directly and the apoptosis regulator gene BCL-W indirectly [81]. miR-409-3p was found to be down-regulated in human gastric tumours, and its expression was significantly associated with the tumour node metastasis stage. An increase in miR-409-3p reduced the migration and invasion of cancer cells *in vitro*, most likely via the pro-metastatic gene radixin (RDX) [82] and the PHF10 gene [83].

Down-regulation of miR-155 was observed in gastric cancer cell lines. miR-155 might act as a tumour suppressor through the repression of its target gene SMAD2, which encodes a signal transducer and transcription modulator protein involved in multiple signalling pathways [84]. Similarly, the down-regulation of miR-148a was observed in gastric tumour tissues, with miR-148a acting as a tumour metastasis suppressor in gastric cancer through the repression of the gene ROCK1, which encodes a serine/threonine kinase involved in the regulation of cell proliferation and programmed cell death [85], and through the repression of the p27 or CDKN1B gene, which encodes an inhibitor protein of the cell division cycle [86]. miR-182 was also found to be significantly down-regulated in human gastric adenocarcinoma tissues, and the over-expression of miR-182 would suppress the proliferation and colony formation of gastric tumours by targeting the oncogene cAMP-responsive element binding protein 1 (CREB1) [87].

The exploration of deregulated miRNAs in gastrointestinal tumours could contribute essential data for the possible development of novel cancer gene therapies [104].

### 4.5. Hepatocellular Cancers

The over-expression of miR-519d was found to have an oncogenic role in hepatocellular carcinoma (HCC) with promoting effects on cell proliferation, invasion and apoptotic impairment by directly targeting the tumour suppressor gene PTEN, insulin regulating gene AKT3 and endothelial suppressing gene TIMP2 [88]. In addition, the down-regulation of miR-519d was demonstrated to have a tumour-suppressive role in the human HCC cell line QGY-7703 through its action on the target gene MKI67, which is associated with the ribosomal RNA transcription necessary for cellular proliferation [92]. miR-373 was up-regulated in human HCC tissue, and protein phosphatase 6 catalytic subunit (PPP6C), acting as a negative cell cycle regulator, was identified as the target gene of miR-373 [89]. In addition, miR-199a was significantly down-regulated in HCC tissues and a few HCC cell lines, including SMMC-7721, BEL-7402 and HepG2, most likely through its effect on the target gene HIF-1α, which is associated with cell growth under low oxygen conditions [90]. Down-regulated miR-124 was reported in HCC tissues and was found to be associated with more aggressive HCC in patients with a poor prognostic phenotype. miR-124 likely targets ROCK2 and EZH2, which are key modulators of cytoskeletal rearrangement and the maintenance of the transcriptional repressive state over successive cell generations, respectively [91].

### 4.6. Other Cancers

miR-330 was found to act post-transcriptionally by regulating deoxycytidine kinase (DCK) mRNA expression, which is essential for the phosphorylation of natural deoxyribonucleosides and their nucleoside analogues, which are widely used as anticancer compounds, including gemcitabine and cytarabine [93]. Elevated miR-330 would suppress DCK expression, leading to the undermined sensitivity to gemcitabine.

**Figure 2:**
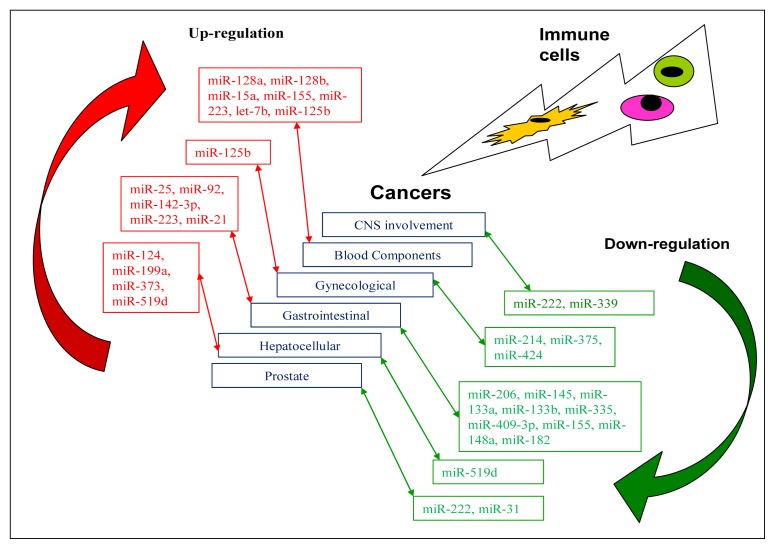
Tumour types and their miRNA expression.

miRNAs are involved in the pathogenesis of prostate cancer, and their roles as oncogenes, tumour suppressors and metastasis regulators lead to the hope that they might be molecularly targeted as diagnostic or prognostic markers for the treatment of prostate cancer [105]. Fuse *et al.*[94] demonstrated that miR-222 and miR-31 inhibited cell proliferation, invasion and migration in the prostate cancer cell lines PC3 and DU145, indicating that these two miRNAs might act as tumour suppressors in prostate cancer. miR-155 is a member of oncomir class of miRNAs and implicated in a wide variety of tumours including colon cancer [95]. Figure 2 signifies the regulated miRNA expressions in various tumour types which might be therapeutically instituted in the arms of immune system.

## 5. Discussion

Emerging evidence has shown that miRNAs are important modulators in cancer pathogenesis within the bigger picture of how cells are transformed into malignant cells and multiply in an uncontrolled manner, followed by tissue invasion and metastasis. Equally, research has also indicated that miRNAs can be effective inhibitors as anti-tumour agents. In this regard, an example such as the antisense-based inhibition of a specific miRNA has been found to be useful due to the enhancement of the corresponding anti-tumour immunity [18].

The development of plasma-circulating miRNA detection and expression profiles, which have been found in association with a range of tumour types, can also be used in therapeutic strategies [103,106] and in various clinical settings of cancer management [107]. As shown in Table 3, a variety of miRNAs are either up-regulated or down-regulated in different tumour types. Examples include the following: oesophageal squamous cell carcinoma identified with the elevated expression of miR-21, miR-25, miR-92, and miR-142-3p; gastric cancer with the down-regulated expression of miR-155, miR-148a, miR-182 and miR-409-3p; and hepatic cellular cancer with the up-regulated expression of miR-124, miR-199a, miR-373 and miR-519d.

## 6. Current Studies Targeting miRNAs for Therapeutic Purposes

Mechanisms of resistance in anticancer treatment have been postulated to be associated with the altered expression of the ATP-binding cassette family of transporters involved in cell membrane transportation. Thus, the emerging role of miRNAs as key gene expression regulators in drug resistance can be the specific mechanism involved in combating the resistance to tyrosine kinase inhibitors in chronic myeloid leukaemia [108]. An understanding of how the involved miRNAs influence the phosphatidylinositol 3 kinase PI3K/AKT signalling pathway in modulating the function of the breast cancer-resistant protein can lead to a therapeutic benefit in breast cancer [109,110].

Techniques that restore the activity of tumour suppressor miRNAs by the inhibition of oncogenic miRNAs using single-stranded antisense oligonucleotides or antimiR have been recently employed for the development of miRNA-based cancer therapeutics [111–113]. In recent studies, certain cancers exhibited dependence on the expression of a single oncogenic miRNA or oncomir [114]. The possible programming of the balance between the expression of oncogenic miRNAs and tumour suppressor miRNAs may result in the specific anti-tumour effect [97].

To target miRNAs in cancer, one strategy involves hindering the oncomir from expression or rebuilding the corresponding tumour suppressor miRNA that might have lost in the cancer. The apoptosis of leukaemic MEG01 could be induced through the reintroduction of miR-15a and miR-16-1, which were shown to inhibit tumour growth *in vivo* in a xenograft model [115], and silencing the oncogenic miR-21 via antisense oligonucleotides has generated an anti-proliferative response *in vitro* in a number of cellular models [116]. Although chemically modified anti-miRNA oligonucleotides have been developed [117,118], nevertheless, their effective delivery into target tissues remains a limitation and needs to be further evaluated for a more specific delivery method with fewer side effects. In addition, the modulation of the miRNA expression via drugs or other agents during their transcription might show the potential of miRNAs as therapeutic adjuvant tools to improve the response and overcome resistance [119].

## 7. Future Challenges

The quantification of extracellular miRNAs in the blood circulation of both healthy and diseased patients was discovered to be confined to the lipid or lipoprotein complexes, such as microvesicles, exosomes or apoptotic bodies, which were highly stable [120]. These circulating miRNAs in cancer patients may serve as a novel diagnostic marker, although their logistic mechanism and the meaning of the quantified signatories of these extracellular miRNAs remain unclear. Hopefully, such identified molecular markers for the prediction of treatment outcome will be very useful, with the expression of circulating miRNAs being used to determine the clinical outcome in cancer patients treated with adjuvant chemotherapy [69].

Different studies have reported conflicting findings or inconsistencies regarding miRNAs from the same tumour, as shown in Table 3; for example, miR-519d has been found to be up-regulated in HCC [88] but down-regulated in a HCC cell line [92]. These findings show that there is a strong need to establish endogenous miRNA controls for normalisation in various methodologies during extraction and quantification. Furthermore, designing a well-planned and controlled analysis of miRNAs in a large cohort of both patients and healthy subjects may be necessary to provide more meaningful evidence for the quantification of miRNA expression and an insightful understanding concerning immunity and tumour biology. Such a trial will eventually lead to clinical advancements in cancer therapy and the significant enhancement of the management of various malignancies.

## Figures and Tables

**Table 1:** miRNAs involved in innate immunity.

		miRNAs	Target gene	Reference
Innate				
	Macrophages	miR-511-3p	MRC1	[13]
		miR-146a	TRAF6, IRAK1	[14]
		miR-27b		[15]
		miR-9	NF-κB1	[16]
		miR-222, miR-339	ICAM-1	[17]
		miR-125b	TNF-α	[18]
		miR-16		[19]
	NK cells	miR-378	GZMB	[20]
		miR-30e	PRF	[20]
		miR-27a *	PRF1 & GZMB	[21]
		miR-30c-1 *	HMBOX1	[22]

* indicates an miRNA expressed at *a low level* relative to the miRNA in the opposite arm of a hairpin.

**Table 2:** miRNAs involved in adaptive immunity.

		miRNAs	Target gene	Reference
Adaptive				
B cells	Pro B cells	miR-34a	FOXP1	[25]
		miR-17~92	BIM	[26,27]
		miR-150	c-MYB	[28–30]
	Pre B cells	miR-155	BIC	[31,32]
		miR-155	SHIP & C/EBPβ	[33]
		miR-125b	LIN28A	[34]
	Immature B cells	miR-15a, miR-16-1		[35]
		miR-181a, miR-181b, miR-107, miR-424	PLAG1	[36,37]
	Naïve B cells	miR-17-5p, miR-106a, miR-181b		[38]
		miR-17-5p, miR-127		[39]
		miR-17~92, miR-106a-363, miR-106b-25		[40]
	Mature B cells	miR-155	PU.1	[41]
T cells		miR-135b	FOXO1, STAT6 & GATA3	[42]
		miR-140-5p, miR-409-3p, miR-433-3p, miR-650	ULBP1	[43]
DCs		miR-155, miR-146a, miR-125a-5p		[8]
		miR-155	AGO2, AGO4	[44]
		miR-23b	NOTCH1	[45]
		miR-146a, miR-155, miR-132		[46,47]
		miR-511		[48]

**Table 3:** Tumour types with miRNA expression against gene targets.

Tumour type	Up-regulated	Down-regulated	Brief description of miRNAs	Target genes	References
CNS involvement					
CNS glioblastoma		miR-222miR-339	Possible therapeutic targets	ICAM1	[17,62]
Blood component					
ALL	miR-128a miR-128b		Possible diagnostic markers		[63]
	miR-19		Oncomir	PTEN, BIM	[64]
	miR-15a			BCL2	[65]
	miR-155				[66]
AML	miR-223, let-7b		Possible diagnostic markers		[63]
APL	miR-125b		Potential therapeutic target	BAK1	[67]
Gynaecological					
Breast cancer	miR-125b				[68,69]
Cervical		miR-424	Tumour suppressor	CHK1, p-CHK1	[70]
		miR-375		SP1	[71]
		miR-214		PLEXIN-B1	[72]
Gastrointestinal					
Laryngeal squamous cell carcinoma		miR-206	Tumour suppressor miRNA	VEGF	[73]
Oesophagus squamous cell carcinoma	miR-92			CDH1	[74]
	miR-25			CDH1	[75]
	miR-142-3p		Potential prognostic marker		[76]
	miR-21		Oncomir		[77]
		miR-145, miR133a, miR-133b	Tumour suppressor miRNAs	FSCN1	[78]
Gastric cancer	miR-21			PTEN	[79]
	miR-223			FBXW7/hCDC4	[80]
		miR-335	Metastasis suppressor miRNA	SP1, BCL-W	[81]
		miR-409-3p		RDX	[82]
		miR-409-3p		PHF10	[83]
		miR-155	Tumour suppressor miRNA	SMAD2	[84]
		miR-148a		ROCK1	[85]
		miR-148a		P27 or CDKN1B	[86]
		miR-182	Tumour suppressor miRNA	CREB1	[87]
Hepatocellular cancer (HCC)					
HCC	miR-519d		Oncomir	PTEN, AKT3, TIMP2	[88]
	miR-373			PPP6C	[89]
	miR-199a			HIF-1α	[90]
	miR-124			ROCK2, EZH2	[91]
HCC cell line QGY-7703		miR-519d	Tumour suppressor miRNA	MKI67	[92]
Other cancers					
Lung cancer		miR-330		DCK	[93]
Prostate cancer		miR-222 miR-31	Tumour suppressor miRNAs		[94]
Colon cancer	miR-155		Oncomir		[95]

Abbreviations: Central Nervous System (CNS); Acute Lymphoblastic Leukemia (ALL); Acute Myeloid Leukemia (AML); Acute Promyelocytic Leukemia (APL).

## References

[1] Li S. Q., Chen F. J., Cao X.F. (2012). Distinctive microRNAs in esophageal tumor: Early diagnosis, prognosis judgment, and tumor treatment. Dis. Esophagus.

[2] Cho W.C. (2010). MicroRNAs in cancer—from research to therapy. Biochim. Biophys. Acta.

[3] Cho W.C. (2010). MicroRNAs: Potential biomarkers for cancer diagnosis, prognosis and targets for therapy. Int. J. Biochem. Cell Biol.

[4] Bavan L., Midwood K., Nanchahal J. (2011). microRNA epigenetics. BioDrugs.

[5] Lodish H. F., Zhou B., Liu G. (2008). Micromanagement of the immune system by miRNAs. Immunology.

[6] Sonkoly E., Stahle M., Pivarcsi A. (2008). MicroRNAs and immunity: novel players in the regulation of normal immune function and inflammation. Semin. Cancer Biol.

[7] Asirvatham A. J., Magner W. J., Tomasi T.B. (2009). miRNA regulation of cytokine genes. Cytokine.

[8] Holmstrom K., Pedersen A. W., Claesson M.H. (2010). Identification of a microRNA signature in dendritic cell vaccines for cancer therapy. Hum. Immunol.

[9] O’Neill L.A. (2006). How Toll-Like receptors signal: What we know and what we don’t know. Curr. Opin. Immunol.

[10] Zhou R., O’Hara S. P., Chen X.M. (2011). MicroRNA regulation of innate immune responses in epithelial cells. Cell Mol. Immunol.

[11] Takeuchi O., Akira S, Rescigno M. (2007). Toll-Like receptor signaling. Dendritic Cell Interactions with Bacteria.

[12] Gantier M. P., Stunden H. J., McCoy C.E. (2012). A miR-19 regulon that controls NF-κB signaling. Nucleic Acids Res.

[13] Squadrito M. L., Pucci F., Maqri L. (2012). miR-511–3p modulates genetic programs of tumor-associated macrophages. Cell Rep.

[14] Taganov K. D., Boldin M. P., Chang K.J. (2006). NF-kappaB-Dependent induction of microRNA miR-146, an inhibitor targeted to signalling proteins of innate immune responses. Proc. Natl. Acad. Sci. USA.

[15] Jennewein C., von Knethen A., Schmid T. (2010). MicroRNA-27b contributes to lipopolysaccharidemediated peroxisome proliferator-activated receptor gamma (PPARgamma) mRNA destabilization. J. Biol. Chem.

[16] Bazzoni F., Rossato M., Fabbri M. (2009). Induction and regulatory function of miR-9 in human monocytes and neutrophils exposed to proinflammatory signals. Proc. Natl. Acad. Sci. USA.

[17] Ueda R., Kohanbash G., Sasaki K. (2009). Dicer-Regulated microRNAs 222 and 339 promote resistance of cancer cells to cytotoxic T-lymphocytes by down-regulation of ICAM-1. Proc. Natl. Acad. Sci. USA.

[18] Tili E., Michaille J. J., Cimino A. (2007). Modulation of miR-155 and miR-125b levels following lipopolysaccharide/TNF-alpha stimulation and their possible roles in regulating the response to endotoxin shock. J. Immunol.

[19] Jing Q., Huang S., Guth S. (2005). Involvement of microRNA in AU-rich element-mediated mRNA instability. Cell.

[20] Wang P., Gu Y., Zhang Q. (2012). Identification of resting and Type I IFN-activated human NK cell miRNomes reveals MicroRNA-378 and MicroRNA-30e as negative regulators of NK Cell cytotoxicity. J. Immunol.

[21] Kim T. D., Lee S. U., Yun S. (2011). Human microRNA-27a* targets Prf1 and GzmB expression to regulate NK-cell cytotoxicity. Blood.

[22] Gong J., Liu R., Zhuang R. (2012). miR-30c-1* promotes natural killer cell cytotoxicity against human hepatoma cells by targeting the transcription factor HMBOX1. Cancer Sci.

[23] Mosser D.M. (2003). The many faces of macrophage activation. J. Leukoc. Biol.

[24] Lisnic V. J., Krmpotic A., Jonjic S. (2010). Modulation of natural killer cell activity by virus. Curr. Opin. Microbiol.

[25] Rao D. S., O’Connell R. M., Chaudhuri A.A. (2010). MicroRNA-34a perturbs B lymphocyte development by repressing the forkhead box transcription factor Foxp1. Immunity.

[26] Ventura A., Young A. G., Winslow M.M. (2008). Targeted deletion reveals essential and overlapping functions of the miR-17 through 92 family of miRNA clusters. Cell.

[27] Koralov S. B., Muljo S. A., Galler G.R. (2008). Dicer ablation affects antibody diversity and cell survival in the B lymphocyte lineage. Cell.

[28] Davidson-Moncada J., Papavasiliou F. N., Tam W. (2010). MicroRNAs of the immune system: Roles in inflammation and cancer. Ann. N. Y. Acad. Sci.

[29] Lin Y.C. (2008). C-Myb is an evolutionary conserved miR-150 target and miR-150/c-Myb interaction is important for embryonic development. Mol. Biol. Evol.

[30] Zhou B., Wang S., Mayr C. (2007). miR-150, a microRNA expressed in mature B and T cells, blocks early B cell development when expressed prematurely. Proc. Natl. Acad. Sci. USA.

[31] Baltimore D., Boldin M. P., O’Connell R.M. (2008). MicroRNAs: New regulators of immune cells development and function. Nat. Immunol.

[32] Costinean S., Zanesi N., Pekarsky Y. (2006). Pre-B cell proliferation and lymphoblastic leukemia/high-grade lymphoma in E(mu)-miR-155 transgenic mice. Proc. Natl. Acad. Sci. USA.

[33] Costinean S., Sandhu S. K., Pedersen I.M. (2009). Src homology 2 domain-containing inositol-5-phosphatase and CCAAT enhancer-binding protein beta are targeted by miR-155 in B cells of Emicro-MiR-155 transgenic mice. Blood.

[34] Chaudhuri A. A., So A. Y., Mehta A. (2012). Oncomir miR-125b regulates hematopoiesis by targeting the gene Lin28A. Proc. Natl. Acad. Sci. USA.

[35] Nana-Sinkam S. P., Croce C.M. (2010). MicroRNA in chronic lymphocytic leukemia: Transitioning from laboratory-based investigation to clinical application. Cancer Genet. Cytogenet.

[36] Pallasch C. P., Patz M., Park Y.J. (2009). miRNA deregulation by epigenetic silencing disrupts suppression of the oncogene PLAG1 in chronic lymphocytic leukemia. Blood.

[37] Patz M., Pallasch C. P., Wendtner C.M. (2010). Critical role of microRNAs in chronic lymphocytic leukemia: Overexpression of the oncogene PLAG1 by deregulated miRNAs. Leuk. Lymphoma.

[38] Tan L. P., Wang M., Robertus J.L. (2009). miRNA profiling of B-cell subsets: Specific miRNA profile for germinal center B cells with variation between centroblasts and centrocytes. Lab. Invest.

[39] Robertus J. L., Harms G., Blokzijl T. (2009). Specific expression of miR-17–5p and miR-127 in testicular and central nervous system diffuse large B-cell lymphoma. Mod. Pathol.

[40] Iqbal J., Shen Y., Liu Y. (2012). Genome-Wide miRNA profiling of mantle cell lymphoma reveals a distinct subgroup with poor prognosis. Blood.

[41] Vigorito E., Perks K. L., Abreu-Goodger C. (2007). MicroRNA-155 regulates the generation of immunoglobulin class-switched plasma cells. Immunity.

[42] Matsuyama H., Suzuki H. I., Nishimori H. (2011). miR-135b mediates NPM-ALK-driven oncogenicity and renders IL-17-producing immunophenotype to anaplastic large cell lymphoma. Blood.

[43] Himmelreich H., Mathys A., Wodnar-Filipowicz A. (2011). Post-transcriptional regulation of ULBP1 ligand for the activating immunoreceptor NKG2D involves 3' untranslated region. Hum. Immunol.

[44] Cubillos-Ruiz J. R., Baird J. R., Tesone A.J. (2012). Reprogramming tumor-associated dendritic cells in vivo using miRNA mimetics triggers protective immunity against ovarian cancer. Cancer Res.

[45] Zheng J., Jiang H. Y., Li J. (2012). MicroRNA-23b promotes tolerogenic properties of dendritic cells in vitro through inhibiting Notch1/NF-κB signalling pathways. Allergy.

[46] Turner M. L., Schnorfeil F. M., Brocker T. (2011). MicroRNAs regulate dendritic cell differentiation and function. J. Immunol.

[47] Nahid M. A., Satoh M., Chan E.K. (2011). MicroRNA in TLR signaling and endotoxin tolerance. Cell Mol. Immunol.

[48] Tserel L., Runnel T., Kisand K. (2011). MicroRNA expression profiles of human blood monocyte-derived dendritic cells and macrophages reveal miR-511 as putative positive regulator of Toll-like receptor 4. J. Biol. Chem.

[49] Hoefig K. P., Heissmeyer V. (2008). MicroRNAs grow up in the immune system. Curr. Opin. Immunol.

[50] Basso K., Sumazin P., Morozov P. (2009). Identification of the human mature B cell miRNome. Immunity.

[51] Medina P. P., Nolde M., Slack F.J. (2010). OncomiR addiction in an *in vivo* model of miR-21-induced pre-B-cell lymphoma. Nature.

[52] Jiang B. H., Liu L.Z. (2008). PI3K/PTEN signaling in tumorigenesis and angiogenesis. Biochim. Biophys. Acta.

[53] Hafsi S., Pezzino F. M., Candido S. (2012). Gene alterations in the PI3K/PTEN/AKT pathway as a mechanism of drug-resistance (review). Int. J. Oncol.

[54] Yu K., Shi C., Toral-Barza L. (2010). Beyond rapalog therapy: Preclinical pharmacology and antitumor activity of WYE-1251332, an ATP-competitive and specific inhibitor of mTORC1 and mTORC2. Cancer Res.

[55] Rao E., Jiang C., Ji M. (2012). The miRNA-17-92 cluster mediates chemoresistance and enhances tumor growth in mantle cell lymphoma via PI3K/AKT pathway activation. Leukemia.

[56] Hart D.N. (1997). Dendritic cells: Unique leukocyte populations which control the primary immune response. Blood.

[57] Palucka K., Ueno H., Fay J. (2011). Dendritic cells and immunity against cancer. J. Intern. Med.

[58] Visone R., Croce C.M. (2009). Keynote lecture: MiRNAs and cancer. Am. J. Pathol.

[59] Bonifer C., Bowen D.T. (2010). Epigenetic mechanisms regulating normal and malignant haematopoiesis: New therapeutic targets for clinical medicine. Expert Rev. Mol. Med.

[60] Fabbri M., Croce C. M., Calin G.A. (2009). MicroRNAs in the ontogeny of leukemias and lymphomas. Leuk. Lymphoma.

[61] Cho W.C. (2007). OncomiRs: The discovery and progress of microRNAs in cancers. Mol. Cancer.

[62] Goldhoff P., Rubin J.B. (2010). Dicer and microRNAs regulate glioma immunoresistance. Immunotherapy.

[63] Mi S., Lu J., Sun M. (2007). MicroRNA expression signatures accurately discriminate acute lymphoblastic leukemia from acute myeloid leukemia. Proc. Natl. Acad. Sci. USA.

[64] Mavrakis K. J., Wolfe A. L., Oricchio E. (2010). Genome-Wide RNA-mediated interference screen identifies miR-19 targets in Notch-induced T-cell acute lymphoblastic leukaemia. Nat. Cell Biol.

[65] Cocco C., Canale S., Frasson C. (2010). Interleukin-23 acts as antitumor agent on childhood B-acute lymphoblastic leukemia cells. Blood.

[66] Canale S., Cocco C., Frasson C. (2011). Interleukin-27 inhibits pediatric B-acute lymphoblastic leukemia cell spreading in a preclinical model. Leukemia.

[67] Zhang H., Luo X. Q., Feng D.D. (2011). Upregulation of microRNA-125b contributes to leukemogenesis and increases drug resistance in pediatric acute promyelocytic leukemia. Mol. Cancer.

[68] Zhou M., Liu Z., Zhao Y. (2010). MicroRNA-125b confers the resistance of breast cancer cells to paclitaxel through suppression of pro-apoptotic Bcl-2 antagonist killer 1 (Bak1) expression. J. Biol. Chem.

[69] Wang H., Tan G., Dong L. (2012). Circulating MiR-125b as a marker predicting chemoresistance in breast cancer. PLoS One.

[70] Xu J., Li Y., Wang F (2012). Suppressed miR-424 expression via upregulation of target gene Chk1 contributes to the progression of cervical cancer. Oncogene.

[71] Wang F., Li Y., Zhou J. (2011). miR-375 is down-regulated in squamous cervical cancer and inhibits cell migration and invasion via targeting transcription factor SP1. Am. J. Pathol.

[72] Qiang R., Wang F., Shi L.Y. (2011). Plexin-B1 is a target of miR-214 in cervical cancer and promotes the growth and invasion of HeLa cells. Int. J. Biochem. Cell Biol.

[73] Zhang T., Liu M., Wang C. (2011). Down-regulation of MiR-206 promotes proliferation and invasion of laryngeal cancer by regulating VEGF expression. Anticancer Res.

[74] Chen Z. L., Zhao X. H., Wang J.W. (2011). microRNA-92a promotes lymph node metastasis of human esophageal squamous cell carcinoma via E-cadherin. J. Biom. Chem.

[75] Xu X., Chen Z., Zhao X. (2012). MicroRNA-25 promotes cell migration and invasion in esophageal squamous cell carcinoma. Biochem. Biophys. Res. Commun.

[76] Lin R. J., Xiao D. W., Liao L.D. (2012). MiR-142–3p as a potential prognostic biomarker for esophageal squamous cell carcinoma. J. Surg. Oncol.

[77] Kimura S., Naqanuma S., Susuki D. (2010). Expression of microRNAs in squamous cell carcinoma of human head and neck and the esophagus: miR-205 and miR-21 are specific markers for HNSCC and ESCC. Oncol. Rep.

[78] Kano M., Seki N., Kikkawa N. (2010). miR-145, miR-133a and miR-133b: Tumor-suppressive miRNAs target FSCN1 in esophageal squamous cell carcinoma. Int. J. Cancer.

[79] Zhang B. G., Li J. F., Yu B.Q. (2012). MicroRNA-21 promotes tumor proliferation and invasion in gastric cancer by targeting PTEN. Oncol. Rep.

[80] Li J., Guo Y., Liang X. (2012). MicroRNA-223 functions as an oncogene in human gastric cancer by targeting FBXW7/hCdc4. J. Cancer Res. Clin. Oncol.

[81] Xu Y., Zhao F., Wang Z. (2012). MicroRNA-335 acts as a metastasis suppressor in gastric cancer by targeting Bcl-w and specificity protein 1. Oncogene.

[82] Zheng B., Liang L., Huang S (2011). MicroRNA-409 suppresses tumour cell invasion and metastasis by directly targeting radixin in gastric cancers. Oncogene.

[83] Li C., Nie H., Wang M. (2012). MicroRNA-409–3p regulates cell proliferation and apoptosis by targeting PHF10 in gastric cancer. Cancer Lett.

[84] Li C. L., Nie H., Wang M. (2012). MicroRNA-155 is downregulated in gastric cancer cells and involved in cell metastasis. Oncol. Rep.

[85] Zheng B., Liang L., Wang C. (2011). MicroRNA-148a suppresses tumor cell invasion and metastasis by downregulating ROCK1 in gastric cancer. Clin. Cancer Res.

[86] Guo S. L., Peng Z., Yang X. (2011). MiR-148a promoted cell proliferation by targeting p27 in gastric cancer cells. Int. J. Biol. Sci.

[87] Kong W. Q., Bai R., Liu T. (2012). MicroRNA-182 targets cAMP-responsive element-binding protein 1 and suppresses cell growth in human gastric adenocarcinoma. FEBS J.

[88] Fornari F., Milazzo M., Chieco P (2012). In hepatocellular carcinoma miR-519d is up-regulated by p53 and DNA hypomethylation and targets CDKN1A/p21, PTEN, AKT3 and TIMP2. J. Pathol..

[89] Wu N., Liu X., Xu X. (2011). MicroRNA-373, a new regulator of protein phosphatase 6, functions as an oncogene in hepatocellular carcinoma. FEBS. J.

[90] Jia X. Q., Cheng H. Q., Qian X. (2012). Lentivirus-mediated overexpression of microRNA-199a inhibits cell proliferation of human hepatocellular carcinoma. Cell Biochem. Biophys.

[91] Zheng F., Liao Y. J., Cai M.Y. (2012). The putative tumour suppressor microRNA-124 modulates hepatocellular carcinoma cell aggressiveness by repressing ROCK2 and EZH2. Gut.

[92] Hou Y. Y., Cao W. W., Li L. (2011). MicroRNA-519d targets MKi67 and suppresses cell growth in the hepatocellular carcinoma cell line QGY-7703. Cancer Lett.

[93] Hodzic J., Giovannetti E., Calvo B.D. (2011). Regulation of deoxycytidine kinase expression and sensitivity to gemcitabine by microRNA-330 and promoter methylation in cancer cells. Nucleosides Nucleotides Nucleic Acid.

[94] Fuse M., Kojima S., Enokida H (2012). Tumor suppressive microRNAs (miR-222 and miR-31) regulate molecular pathways based on miRNA expression signature in prostate cancer. J. Hum. Genet..

[95] Yin Q., Wang X., Fewell C. (2010). MicroRNA miR-155 inhibits bone morphogenetic protein (BMP) signaling and BMP-mediated Epstein-Barr virus reactivation. J. Virol.

[96] Ghiringhelli H., Rebe C., Hichami A. (2012). Immunomodulation and anti-inflammatory roles of polyphenols as anticancer agents. Anitcancer Agents Med. Chem.

[97] Iorio M. V., Croce C.M. (2012). MicroRNA dysregulation in cancer: Diagnostics, monitoring and therapeutics. A comprehensive review. EMBO Mol. Med.

[98] Lin Z., Flemington E.K. (2010). miRNAs in pathogenesis of oncogenic human viruses. Cancer Lett.

[99] Yanaihara N., Caplen N., Bowman E. (2006). Unique microRNA molecular profiles in lung cancer diagnosis and prognosis. Cancer Cell.

[100] Schotte D., De Menezes R. X., Akbari Moqadam F. (2011). MicroRNAs characterize genetic diversity and drug resistance in pediatric acute lymphoblastic leukemia. Haematologica.

[101] Zhang H., Luo X. Q., Zhang P. (2009). MicroRNA patterns associated with clinical prognostic parameters and CNS relapse prediction in pediatric acute leukemia. PLoS One.

[102] Cocco C., Airoldi I. (2011). Cytokines and microRNA in pediatric B-acute lymphoblastic leukemia. Cytokine Growth Factor Rev.

[103] Gilabert-Estelles J., Braza-Boils A., Ramon L.A. (2012). Role of microRNAs in gynecological pathology. Curr. Med. Chem.

[104] Tasawa H., Kagawa S., Fujiwara T. (2011). MicroRNAs as potential target gene in cancer gene therapy of gastrointestinal tumors. Expert Opin. Biol. Ther.

[105] Gordonpour A., Nam R. K., Sugar L (2012). MicroRNAs in prostate cancer: From biomarkers to molecularly-based therapeutics. Prostate Cancer Prostatic Dis..

[106] Cho W.C. (2011). Circulating microRNAs as minimally invasive biomarkers for cancer theragnosis and prognosis. Front. Genet.

[107] Corsini L. R., Bronte G., Terrasi M. (2012). The role of microRNAs in cancer: Diagnostic and prognostic biomarkers and targets of therapies. Experts Opin. Ther. Targets.

[108] Rodriques A. S., Dinis J., Gromicho M. (2012). Genomics and cancer drug resistance. Curr. Pharm. Biotechnol.

[109] Nakanishi T., Ross D.D. (2012). Breast cancer resistance protein (BCRP/ABCG2): Its role in multidrug resistance and regulation of its gene expression. Clin. J. Cancer.

[110] Natarajan K., Xie Y., Baer M.R. (2012). Role of breast cancer resistance protein (BCRP/ABCG2) in cancer drug resistance. Biochem. Pharmacol.

[111] Davis S., Lollo B., Freier S. (2006). Improved targeting of miRNA with antisense oligonucleotides. Nucleic Acid Res.

[112] Stenvang J., Petri A., Lindow M. (2012). Inhibition of microRNA function by antimiR oligonucleotides. Silence.

[113] Thorsen S. B., Obad S., Jensen N.F. (2012). The therapeutic potential of MicroRNAs in cancer. Cancer J.

[114] Cheng C. J., Slack F.J. (2012). The duality of OncomiR addiction in the maintenance and treatment of cancer. Cancer J.

[115] Calin G. A., Cimmino A., Fabbri M. (2008). MiR-15a and miR-16–1 cluster functions in human leukemia. Proc. Natl. Acad. Sci. USA.

[116] Si M. L., Zhu S., Wu H. (2007). miR-21-mediated tumor growth. Oncogene.

[117] Krutzfeldt J., Rajewsky N., Braich R. (2005). Silencing of microRNAs *in vivo* with ‘antagomirs’. Nature.

[118] Weiler J., Hunziker J., Hall J. (2006). Anti-miRNA oligonucleotides (AMOs): Ammunition to target miRNAs implicated in human disease?. Gene Ther.

[119] Chen F., Zhu H. H., Zhou L.F. (2010). Inhibition of c-FLIP expression by miR-512–3p contributes to taxol-induced apoptosis in hepatocellular carcinoma cells. Oncol. Rep.

[120] Kosaka N., Lguchi H., Ochiya T. (2010). Circulating microRNA in body fluid: A new potential biomarker for cancer diagnosis and prognosis. Cancer Sci.

